# Increased labor induction and women presenting with decreased or altered fetal movements - a population-based survey

**DOI:** 10.1371/journal.pone.0216216

**Published:** 2019-05-02

**Authors:** Anna Akselsson, Helena Lindgren, Susanne Georgsson, Karin Pettersson, Ingela Rådestad

**Affiliations:** 1 Sophiahemmet University, Stockholm, Sweden; 2 Department of Women and Children´s Health, Karolinska Institute, Stockholm, Sweden; 3 Department of Clinical Science, Intervention and Technology, Karolinska Institute, Stockholm, Sweden; 4 The Swedish Red Cross University College, Stockholm, Sweden; PreTel, UNITED STATES

## Abstract

**Introduction:**

Women’s awareness of fetal movements is important as perception of decreased fetal movements can be a sign of a compromised fetus. We aimed to study rate of labor induction in relation to number of times women seek care due to decreased or altered fetal movements during their pregnancy compared to women not seeking such care. Further, we investigated the indication of induction.

**Material and methods:**

A prospective population-based cohort study including all obstetric clinics in Stockholm, Sweden. Questionnaires were distributed to women who sought care due to decreased or altered fetal movements ≥ 28 week’s gestation in 2014, women for whom an examination did not indicate a compromised fetus that required induction of labor or cesarean section when they sought care. Women who gave birth at ≥ 28 weeks’ gestation in 2014 in Stockholm comprises the reference group.

**Results:**

Labor was induced more often among the 2683 women who had sought care due to decreased or altered fetal movements (RR 1.4, 95% CI 1.3–1.5). In women who presented with decreased or altered fetal movements induction of labor occurred more frequently for fetal indication than those with induction of labor and no prior fetal movement presentation (RR 1.6, 95% CI 1.4–1.8). The rate of induction increased with number of times a woman sought care, RR 1.3 for single presentation to 3.2 for five or more.

**Conclusions:**

We studied women seeking care for decreased or altered fetal movements and for whom pregnancy was not terminated with induction or caesarean section. Subsequent (median 20 days), induction of labor and induction for fetal indications were more frequent in this group compared to the group of women with no fetal movement presentations. Among women seeking care for altered or decreased fetal movements, the likelihood of induction of labor increased with frequency of presentation.

## Introduction

Fetal movements are an important indication of wellbeing in the unborn baby [1–3]. Decreased fetal movements can be a sign of a vulnerable fetus, a prolonged reaction due to chronic hypoxia [3–9]. Increased maternal awareness of fetal movements is associated with reduction of perinatal mortality and the mother’s perception of fetal movements is considered as a valuable screening tool [2]. Further, low awareness of fetal movements is associated with children born small for gestational age [10]. Though, Walker and Thornton [11] recently argued in *The Lancet*; “Discouraging campaigns that promote awareness preterm, improving induction guidelines, and not inducing delivery in response to perception of altered movement alone would seem to be sensible first steps.” The authors suggest that professional health-care providers should not encourage women to be aware of fetal movements. This suggestion is based on the results of the AFFIRM study [12], where the stillbirth incidence was lowered in the intervention group (from 4.40/1000 to 4.06/1000), although the difference was not statistically significant, while the rates of induction of labor and cesarean section were higher in the intervention group. However, the intervention did include both encouraging women to be aware of fetal movements and changes in management when the women presented with decreased fetal movements. As Flenady and co-authors [13] suggest in their reply to Walker and Thornton [11], in cases of unnecessary interventions, the outcome might very well depend on the dysfunctional management of the professional caregivers as much as women being aware of fetal movements. Nevertheless, there is no consensus as to what extent professional caregivers should encourage women to be aware of fetal movements.

Concerns about decreased fetal movements are common reasons for women to make unplanned visits to an antenatal clinic [14,15]. Between six and 15 percent of all pregnant women seek care due to decreased or altered fetal movements during the third trimester [16–18]. Of those who sought care, Sinha et al. [7] found that 71 percent sought care one time, 23 percent two times and four percent three times or more. In some of the cases, where women seek care due to decreased fetal movements, a compromised fetus or intrauterine fetal death is detected [19]. However, for the majority of the women no abnormality is found and the women can after an examination of the fetus, go home [18].

The routine practice for midwives in primary care, is to refer women who perceive decreased fetal movements to the obstetric clinic for further investigation. According to The Swedish National Board of Health and Welfare [20], the evaluation should include a detailed fetal movement history from the woman and examination by cardiotocography (CTG). Individualized assessment and decision making determine need for further investigations such as ultrasound. Every hospital has local management guidelines of women presenting with decreased fetal movements. The guidelines of The Royal College of Obstetricians and Gynecologists and the National Board of Health and Welfare [21] state that women should be advised to visit healthcare again if they perceive a new episode of decreased fetal movements after having experienced at least one such episode.

Women who seek care due to decreased fetal movements have their labor induced to a higher extent than women who don’t seek such care. In studies from Ireland and Malaysia [22,23] the induction rate is 50 percent higher among women who present with decreased fetal movements. There is no doubt that induction of labor improves pregnancy outcome in comparison to expectant management after gestation week 40; based on 30 randomized clinical trials included in a Cochrane review [24] (the majority had induction after ≥41 weeks’ gestation), the risk ratio of perinatal death was 0.33 (95% CI 0.11–0.96). Further, the number of cesarean sections, rates of NICU admissions, and number of babies with Apgar scores less than seven, were lower for women in the policy of induction compared to expectant management. Induction before gestation week 40, is, for every woman, a balance between the risk of unnecessary intervention and a window of opportunity to prevent an adverse pregnancy outcome and we lack in evidence for forming a policy.

As far as we know, no data on rate of induction among women who seek care due to decreased or altered fetal movements in Sweden is available. Further, there is a gap of knowledge about the rate of induction among women who seek care due to decreased fetal movements for whom an examination does not indicate a compromised fetus that required induction of labor when they seek care.

When a woman presents with decreased fetal movements repeatedly it can be an indicator that she is continuously aware of the movements, which in turn can lead to reduced pre-hospital delay if the fetus is at risk. To understand the effect of women’s awareness of fetal movements, we studied women seeking care for decreased or altered fetal movements and for whom pregnancy was not terminated with induction or caesarean section; these women completed a questionnaire. After a median duration of 20 days (inter-quartile range 6–42 days), the women gave birth, and we then obtained information about pregnancy outcomes from the population-based register, Obstetrix. As a comparison group, we chose women without presentation for decreased fetal movements.

## Material and methods

We carried out a population-based prospective study in Stockholm County. A questionnaire was handed out at obstetric clinics to women who sought care due to decreased or altered fetal movements from January 1, 2014 to December 31, 2014, whose pregnancy was not terminated with induction or caesarean section, when they sought care. All seven obstetric clinics in Stockholm were included; inclusion criteria were women ≥ 28 weeks’ gestation with a singleton pregnancy.

*The questionnaire* was validated face-to-face with ten pregnant women before the main data collection began. It comprises 22 questions asking about fetal movements and the woman’s background (see supporting files, S1 File and S2 File, which includes questionnaires in English and in Swedish). The questionnaire was available in Swedish, English, Spanish, Arabic, Sorani, Farsi, and Somali. The question, asking about the number of times the woman had sought care due to decreased fetal movements was formulated as: “How many times previously (during your present pregnancy) have you sought medical care because you expected baby has moved less or differently?” The women could choose a response ranging from ‘never’ to ‘four times or more’.

### Outcome assessment

Information concerning pregnancy outcome was retrieved from the population-based pregnancy and birth register (Obstetrix). The link to the information in the questionnaire was made by the unique personal identity number assigned to each Swedish resident.

### Reference group

Women with singleton pregnancies who gave birth ≥ 28 weeks’ gestation, during the period from January 1 to December 31, 2014 at any one of Stockholm’s seven obstetric clinics were eligible for inclusion in the reference group. Identification of the women in the reference group was carried out by using the population-based pregnancy and birth register (Obstetrix). By using the personal identity number in the completed questionnaires, we excluded women who had sought care for decreased fetal movements from the reference group.

### Obstetric routines

The guidelines regarding management of women presenting with decreased fetal movements are local and might differ between hospitals. The National Board of Health and Welfare [20] recommend an investigation, including a detailed fetal movement history obtained from the woman, and examination by cardiotocography. Individual judgement decides whether further investigations are needed. Ultrasound can be used to measure amniotic fluid, growth, fetal movements and blood flow. If a woman during pregnancy contacts her local maternity clinic with concerns about decreased fetal movements, the recommendation is that she attends the obstetric clinic at the hospital for an examination. At the time for data collection, no routines were present in management guidelines for diagnostic coding of decreased fetal movements for the medical staff and these data were only collected from the questionnaires.

*Statistical analyses* were carried out using SPSS version 24.0.0.0 (IBM, SPSS Statistics). We calculated average, frequency, and percentage for the different variables. To analyze differences in socio-economic factors and pregnancy outcomes between groups we used Fisher’s exact test (two-tailed). Calculation of prevalence ratios (sited as relative risk, RR) and 95 percent confidence intervals were based on binomial distribution. Statistically significant differences between the groups that were compared were defined at the 5% level. Induction of labor was adjusted for possible confounding factors (BMI, parity, education, assisted conception, country of birth, and age) by logistic regression. We also determined fetal indications for induction of labor using the following ICD-10 [25] diagnostic codes: Signs of hypoxia (O363), Known or suspected intrauterine growth retardation (O365), Known or suspected specified problems of the fetus (O368), Other specified (suspected) problems of the fetus (fetal heart arrhythmia) (O368B), Known or suspected problems of the fetus (O368W), Known or suspected problem of fetus, unspecified (O369) and Oligohydramnios (O410).

The study was approved by The Regional Ethic committee in Stockholm, Sweden, approval number 2013/1077-31/3.

## Results

**Fig 1:**
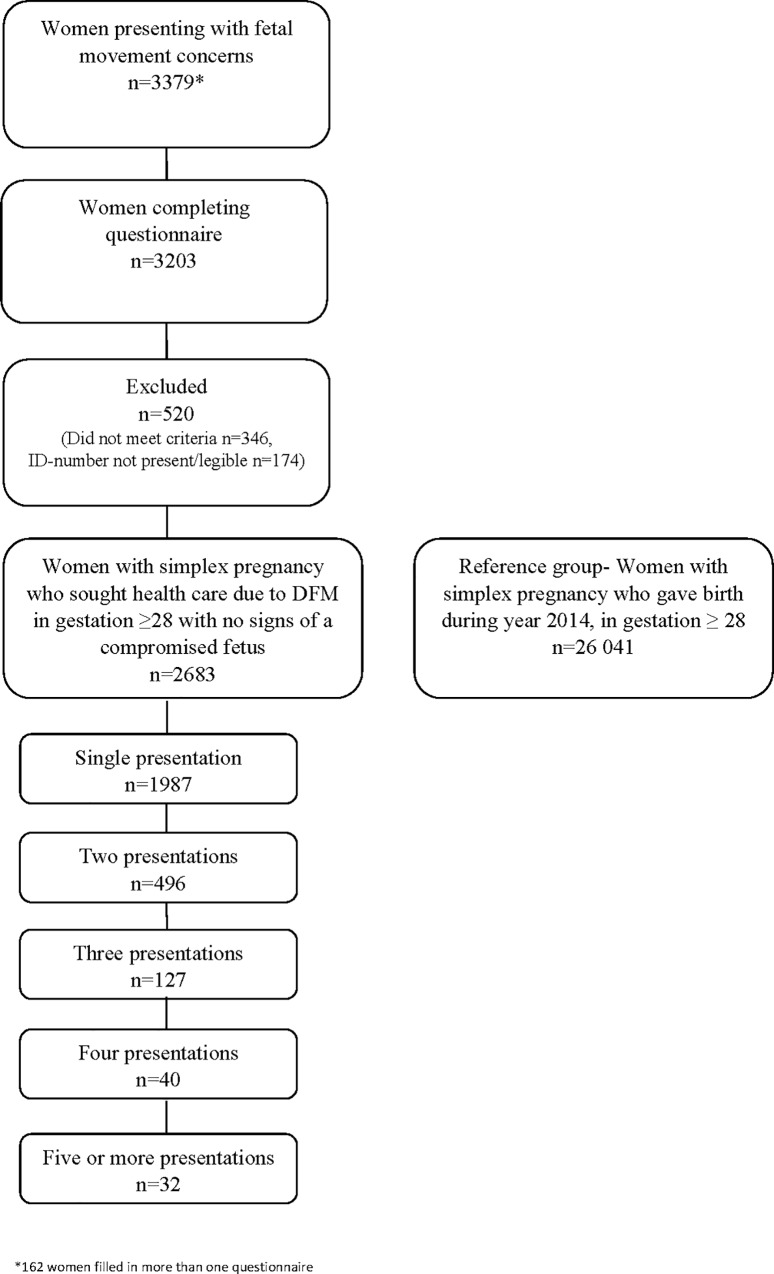
Flow chart describing the study population.

A total of 3541 questionnaires were received, by hand to hospital staff at time of visit (n = 3341) or by post (n = 38). For women completing multiple questionnaires (n = 162), only the most recent was used in the analysis. As shown in Fig 1, 176 women did not submit a complete questionnaire, 346 women did not meet the inclusion criteria and 174 women had not given a complete Swedish personal identity number (or it was unclear written) in the questionnaire. Data from 2683 women who sought care due to decreased or altered fetal movements and without an immediate indication for delivery are included in the analyses. In the reference group we have data from 26041 women.

The women who had presented with decreased fetal movements sought care during gestational week 28+0 to 42+2, average 36+2, and gave birth between gestational week 30+2 to 42+3, average 39+4. The women gave birth after a median duration of 20 days (inter-quartile range 6–42 days) from when they sought care. The women in the reference group gave birth between gestational week 28+0 and 44+6, average 39+2. Gestation calculated from ultrasound. Compared to the reference group, the women who presented with decreased or altered fetal movements were younger, more often nulliparous, born in Sweden, had a higher level of education, had higher BMI and were more likely to have become pregnant after in vitro fertilization (Table 1).

**Table 1:** Characteristics for women who sought care due to decreased or altered fetal movements (DFM group) and women who gave birth in 2014 (Reference group).

Characteristics	Women in DFM group n = 2683 n (%)	Reference group n = 26041 n (%)	p-value^1^
**Maternal age (years)**^**2**^			
≤ 19–24	228 (8.5)	1802 (7.0)	0.005
25–34	1723 (64.2)	15821 (61.4)	0.004
35–39	562 (20.9)	6266 (24.3)	<0.001
≥ 40	170 (6.3)	1872 (7.3)	0.077
**Country of birth**^**3**^			
Sweden	1788 (77.5)	16151 (70.9)	<0.001
Other Nordic countries	31 (1.3)	242 (1.1)	0.207
Rest of Europe	154 (6.7)	2042 (9.0)	<0.001
Asia	200 (8.7)	2400 (10.5)	0.005
Africa	74 (3.2)	1310 (5.8)	<0.001
South America	34 (1.5)	374 (1.6)	0.604
North America/Canada	10 (0.4)	129 (0.6)	0.554
Other countries	17 (0.7)	126 (0.6)	0.247
**Level of Education**^**4**^			
Schooling < 9 years	3 (0.1)	119 (0.6)	0.005
Primary school or equivalent	75 (3.6)	1259 (6.4)	<0.001
Up to and including upper secondary school or equivalent	648 (31.5)	5882 (29.8)	0.111
University; college or equivalent	1334 (64.8)	12502 (63.3)	0.186
**Parity**			
Nulliparous	1431 (53.3)	11747 (45.1)	<0.001
Multiparous	1252 (46.7)	14294 (54.9)	<0.001
**Conception**			
Assisted conception	155 (5.8)	1213 (4.7)	0.012
Spontaneous conception	2528 (94.2)	24828 (95.3)	0.012
**Smoking in early pregnancy**			
Smoking	97 (3.6)	993 (3.8)	0.671
Non-smoker	2586 (96.4)	25048 (96.2)	0.671
**Body Mass Index (kg/m**^**2)**^ ^**5**^			
<18.5	57 (2.2)	789 (3.1)	0.007
18.5–24.9	1642 (62.2)	16772 (65.7)	<0.001
25.0–29.9	633 (24.0)	5608 (22.0)	0.019
30.0–34.9	240 (9.1)	1757 (6.9)	<0.001
35.0–39.9	54 (2.0)	471 (1.8)	0.450
≥40.0	14 (0.5)	121 (0.5)	0.657

^1^ Fisher´s exact test.

^2^ Missing = 280 in reference group.

^3^ Missing = 3642 in total; 375 in DFM group and 3267 in reference group.

^4^ Missing = 6902 in total; 623 in DFM group and 6279 in reference group.

^5^ Missing = 566 in total; 43 in DFM group and 523 in reference group.

As shown in Table 2, 1987 (74%) women sought care on one occasion, 496 (18.5%) two times, 127 (4.7%) three times, 40 (1.5%) four times and 32 (1.2%) five times or more. Among women who sought care due to decreased fetal movements labor was induced in 672 (25.1%) cases compared with 4525 (17.4%) of the women in the reference group (RR 1.4 95% CI 1.3–1.5). The corresponding figures for women who sought care one time were 1.3 (95% CI 1.2–1.4), two times 1.6 (95% CI 1.4–1.9), three times 1.9 (95% CI 1.5–2.4), four times 2.9 (95% CI 2.1–3.9), and five times or more 3.2 (95% CI 2.4–4.4). The percentage of women who had a cesarean section prior to the onset of labor was lower among women who had sought care due to decreased or altered fetal movements; 10.5 percent compared to 12.2 percent (RR 0.9 95% CI 0.77–0.97) in the reference group.

**Table 2:** Pregnancy outcomes of women who gave birth in 2014 (Reference group) and women who sought care due to decreased or altered fetal movements (DFM group) divided after number of occasions the women sought care.

Outcome	Reference group n = 26041 n (%)	Sought care due to DFM one time n = 1987 n (%)	Sought care due to DFM two times n = 496 n (%)	Sought care due to DFM three times n = 127 n (%)	Sought care due to DFM four times n = 40 n (%)	Sought care due to DFM ≥five times n = 32 n (%)
**Onset of labor**^**1**^						
Spontaneous	18351 (70.5)	1322 (66.5)	310 (62.5)	70 (55.1)	17 (42.5)	10 (31.3)
Induction	4525 (17.4)	453 (22.8)	139 (28.0)	42 (33.1)	20 (50.0)	18 (56.3)
Caesarean section before contractions	3165 (12.2)	212 (10.7)	47 (9.5)	15 (11.8)	3 (7.5)	4 (12.5)
**Caesarean section in spontaneous onset of labor**^**2**^						
No	17004 (92.6)	1219 (92.2)	281 (90.6)	64 (91.4)	14 (82.4)	9 (90.0)
Yes	1347 (7.3)	103 (7.8)	29 (9.4)	6 (8.6)	3 (17.6)	1 (10.0)

^1^ Onset of labor missing: reference group 0/ DFM group 1

^2^ Proportion of women whose delivery started spontaneously

When we compare women, who have presented with decreased or altered fetal movements with our reference group according to the outcome induction of labor, and adjust for BMI, parity, education, assisted conception, country of birth and age, one single variable at a time, the risk ratio does not change, or it increases from 1.4 to 1.5 (Table 3).

**Table 3:** Induction of labor among 672 women in the DFM-group in comparison to 4525 women in the reference group with adjustment for possible confounders by logistic regression.

Induction of labor	RR (CI)	P-value
Unadjusted	1.4 (1.3–1.5)	<0.001
Adjusted for:		
BMI (grouped)	1.4 (1.3–1.5)	<0.001
Parity	1.4 (1.3–1.5)	<0.001
Education	1.4 (1.3–1.6)	<0.001
Assisted conception	1.4 (1.3–1.5)	<0.001
Country of birth (grouped-region)	1.5 (1.4–1.6)	<0.001
Age (grouped)	1.5 (1.3–1.6)	<0.001

Missing: BMI; 13 DFM-group, 0 Reference group, Education; 3 DFM-group, 1077 Reference group

Country of birth; 2 DFM-group, Reference group 570, Age; 0 DFM-group, 57 Reference group

For induction indications, 213 of 672 (31.7%) were based on a condition observed in the fetus (Table 4). The corresponding figure for the reference group is 907 of 4525 (20.0%), and the difference is statistically significant (RR 1.6 95% CI 1.4–1.8). The indications given were “Signs of hypoxia”, “Suspected and diagnosed intrauterine fetal growth restriction”, “Oligohydramnios” and “Problems with fetus”.

**Table 4:** Labor induction due to fetal indication among women in the DFM group and women in the reference group.

Labor induction due to fetal indication*	Labor induction among women in DFM group n = 672 n (%)	Labor induction among women in reference group n = 4525 n (%)	RR (CI)	P-value
Signs of hypoxia (O363)	7 (1.0)	79 (1.7)	0.60 (0.28–1.29)	0.25
Suspected and diagnosed intrauterine fetal growth restriction (O365)	78 (11.6)	419 (9.3)	1.25 (1.00–1.57)	0.06
Oligohydramnios (O410)	56 (8.9)	286 (6.8)	1.32 (1.00–1.74)	0.05
Problems with fetus (O368, O368B, O368W, O369)	72 (10.7)	123 (2.7)	3.9 (3.00–5.21)	<0.001
**Total**	**213 (31.7)**	**907 (20.0)**	**1.6 (1.4–1.8)**	**<0.001**

* ICD-10

When we compare women, who had presented with decreased fetal movements sometime during pregnancy with the reference group, we found a lower number of babies having an Apgar score below seven at five minutes, lower rate of preterm labor, and a lower number of babies transferred to neonatal nursery (Table 5). Only the lower rate of preterm labor result was statistically significant.

**Table 5:** Birth outcomes of women in the DFM group in comparison with reference group.

Birth outcome	DFM-group n = 2683 n (%)	Reference group n = 26041 n (%)	RR (CI)	P-value
Apgar Score < 7 at 5 min*	19 (0.7)	283 (1.1)	0.65 (0.41–1.03)	0.07
**Preterm labor**	76 (2.8)	1174 (4.5)	0.63 (0.50–0.79)	<0.001
Birthweight <-22%**	60 (2.2)	503 (1.9)	1.16 (0.89–1.51)	0.27
Transfer to NICU***	78 (2.9)	930 (3.6)	0.81 (0.65–1.02)	0.08

*Stillbirth: 1 (0.03%) in DFM-group (presented once with DFM), 59 (0.2%) in reference group

**Swedish definition of Small for Gestational Age (SGA)

***Neonatal Intensive Care Unit

Missing; Apgar Score: DFM-group = 1 (presented 1 time), Reference group = 87, Preterm birth: DFM-group = 0, Reference group = 0, Birthweight: DFM-group = 1 (presented 1 time), 1 (presented 2 times), 1 (presented 5 times or more), Reference group = 0, NICU: DFM-group = 0, Reference group = 13

## Discussion

In this large-scale population-based setting including all seven obstetric clinics in Stockholm during 2014, we found that the rate of induction of labor increased due to the number of times the woman sought care due to decreased or altered fetal movements. Further, the induction of labor was more often based on fetal indication among women who sought care as compare to women who did not sought care to decreased fetal movements. The number of elective cesarean sections was lower among women who had sought care due to decreased fetal movements. Incidences of preterm labor, Apgar score below seven at five minutes, and transfer to neonatal care were also lower, but only preterm labor was statistically significant.

We found that the induction of labor among the women who sought care due to decreased or altered fetal movements often were based on fetal indication. Neogi et. al. [26] found that a pro-active and timely action in antenatal care can prevent adverse pregnancy outcomes such as stillbirths. Further, induction of labor at term and post-term is associated with a lower percentage of perinatal deaths and cesarean birth compared with expectant management [27]. The women in this study, who sought care due to decreased or altered fetal movements reacted to changes and took action**s** when they had concerns of their unborn baby’s well-being. However, no intervention was done in connection to the examination of the unborn baby when presenting with decreased fetal movements. If the women in the DFM-group had induction of labor or cesarean section it was performed later, at another occasion (median 20 days). Thus, a woman’s perception of decreased or altered fetal movements might indicate that her unborn baby is at risk for compromised health despite no signs of compromise are identified when she seeks care.

It is well known that some women who have experienced stillbirth have waited too long before they contact health care for an examination of their unborn baby [5,28,29]. Linde et al [30] found that a reason for prehospital delay was not wanting to burden health care. Other reasons why women did not want to consult care due to decreased fetal movements were that they did not want to feel that they were annoying or be perceived as excessively worried. Warland et. al. [31] report that some women who have experienced intrauterine fetal death described having a “gut feeling” that something was wrong, sometimes long before the baby died in utero and the authors suggest this maternal intuition should be taken seriously.

The percentage of occasions the women in our study sought care due to decreased or altered fetal movements is similar to the percentage in in a study from United Kingdom performed by Sinha et al. [7]. As far as we know, no studies have been done among women who seek care for decreased fetal movements for whom examination do not indicate a compromised fetus that required induction of labor when they seek care. Earlier research comprises all women seeking care due to decreased fetal movements, i.e. including all women that intervention is done in connection to the visit for decreased fetal movements. In Ireland, 42 percent of all the women who sought care for decreased fetal movements had induced labor compared with 28 percent in the reference group that did not seek care [22]. Furthermore, data from Malaysia also show differences in the occurrence of induction similar to those reported from Ireland. Among those women in Malaysia who sought care, labor was induced in 45 percent [23]. Differences between women who seek health care and other are also shown in Israel where labor was induced in 17 percent of the women who sought care compared with six percent in the reference group [32].

At the time for data collection there were no Swedish national guidelines for maternity clinics specifying what kind of information should be given to pregnant women about fetal movements. However, in October 2016 (almost two years after completed data collection) the Swedish National Board of Health and Welfare formulated guidelines that recommend that all women from gestational week 24 should receive information from their midwife about fetal movements and when to seek health care [20]. We do not know if the women in our study were well informed and had received information when to sought healthcare due to concerns about fetal movements. However, available data do indicate that if a woman is well informed and seeks care when she experiences that fetal movements has changed from an established pattern (decreased or become altered), the outcome of the pregnancy is likely to be improved. The results from a Norwegian study published in 2009 indicate that information given to pregnant women about the importance of seeking care if fetal movements decrease reduced the percentage of stillbirth [33]. Furthermore, the mother’s ability to identify important changes in fetal movement increases the probability that midwife or physician can identify fetuses that are experiencing growth restriction and thus improve perinatal outcomes [34]. In a systematic review from 2016 the researchers conclude that according to available evidence they could not find either benefit or harm in maternal awareness of fetal movements and women should be continued to be informed about the importance of fetal movements for fetal health [35].

We found that women who sought care due to decreased fetal movements were younger and nulliparous, were born in Sweden, had higher level of education, were overweight and had in vitro fertilization more often than women who did not seek care. In the United Kingdom, researchers found that women who sought care for decreased fetal movements were younger than women who did not seek such care [36]. No such differences in age were found in two studies in Norway or Ireland [19,22] but in another Norwegian study it was seen that women 34 years of age or older have a lower awareness of fetal movements than do younger women [10]. Women with low levels of education did not present with decreased fetal movements to the same extent as well educated and this is an important finding. A lower educational level might be expected to be associated with lower levels of knowledge and awareness about fetal movements [37]. According to Zeitlin et al. [38], low educational level is associated with a higher risk for stillbirth, and it may be especially important for professional healthcare providers to be proactive when caring for women in socially vulnerable groups, who have twice the risk of stillbirth in comparison with women in socially advantaged groups [39]. Further, it is of importance to consider special information needs of these women in antenatal care.

The percentage of nulliparous in the women in our study who sought care due to decreased fetal movements was greater than in the reference group. Differences were also observed by McCarthy et al. [22] where half of the women who sought care were nulliparous compared with only one third in the reference group. A similar difference was obtained in the Norwegian study where 51 percent were nulliparous compared with 39 percent in the reference group [19]. The Norwegian study also documented overweight as predicting that a woman would seek care due to decreased fetal movements. This finding was confirmed in a later review where the authors found that women with overweight are more likely to present with decreased fetal movements but could not demonstrate that these women have impaired perception of fetal movements [40]. Women having overweight are a risk factor for stillbirth and a rising problem in high-income countries [41].

It is likely that we have missed an opportunity to distribute questionnaires to some women who met the criteria for presenting with decreased fetal movements. Thus, these women were included in the reference group. Given the close contact between the research team and the seven obstetric clinics, we believe that there were few women who met the inclusion criteria who did not complete the questionnaire. According to the covariation between seeking care due to decreased fetal movements and induction of labor, this source of potential error is expected to lead to a dilution of differences between the groups, i.e., this relationship, in reality, is stronger than our measure of covariation indicates. It is also likely that there are women in the reference group who came to the hospital expressing concerns for decreased fetal movements and then were induced or delivered by cesarean section soon after arrival. In some of these cases, the cause of resulting in a compromised fetus might have been acute onset that could not have been prevented by the woman, for example placental abruption. These women, of course, don’t have the same option to be proactive and seek healthcare at an appropriate or best time. Some women might have sought care after observing decreased fetal movements, but this could have been because their baby had died in utero. In these cases, the women perceived an absence of fetal movements and this group was not studied. It is not clear how much cultural or social differences, or the nature of Swedish healthcare and its guidelines might affect the application of what we have learned to practice in other countries. In a Cochrane review from 2012 [42] the authors conclude that we do not have sufficient data to determine the best management of women presenting with decreased fetal movements. We do not have information about the numbers of women who contact their midwife at the maternity clinic in primary care due to decreased fetal movements who are not referred to hospital for an examination. We expect that the distortion that this source error introduces dilutes the effect, when decreased fetal movements are related to induction of delivery.

Our results indicate that Walker and Thornton’s [11] position that discouraging campaigns that promote awareness of fetal movements may be counterproductive; that is to say, if we followed their advice, the number of babies born with compromised wellbeing or stillborn would increase. Probably the women in our study, those who sought care repeatedly, were often more aware of the fetal movements, and to a higher extent, than those who did not seek care; at the same time, their pregnancy outcomes were better and the presence of elective cesarean sections did not increase. An understanding of the effect of promoting awareness of fetal movements by professional healthcare providers requires randomized intervention trials and, if anything, our study shows that such research is possible to conduct, even after making ethical considerations. Two large studies are ongoing, Mindfetalness (https://clinicaltrials.gov NCT02865759) and My Baby’s Movements Trial (http://www.anzctr.org.au ACTRN12614000291684). Further, it may be especially important for professional healthcare providers to be proactive when caring for women in socially vulnerable groups, where the risk of stillbirth is higher than for the rest of the population. It is possible that this might be investigated in the two ongoing studies.

## Conclusion

We found that women who sought care due to decreased or altered fetal movements sometime during pregnancy were induced more often and that the induction rate increased in relation to the number of times the women sought care. Further, women who presented with decreased fetal movements were induced to a higher extent on the basis of fetal indication.

## Supporting information

S1 File Questionnaire in Swedish language. (PDF)

S2 File Questionnaire in English language. (PDF)
