# Supplementary material for: Increased labor induction and women presenting with decreased or altered fetal movements - a population-based survey
Source: PLoS One. 2019 May 2;14(5):e0216216. doi: 10.1371/journal.pone.0216216 (PMC6497262; doi:10.1371/journal.pone.0216216)
Supplement: S1 File — (PDF) [file pone.0216216.s001.pdf]

Fosterrörelser efter graviditetsvecka 28

1. Söker du vård för att du känt att ditt barn har rört sig **mindre eller annorlunda?**

☐ Ja ☐ Nej, jag söker vård för…………………………………………………

2. Vilket datum är ditt barn beräknat att födas (år-månad-dag)?............................

3. Hur många gånger tidigare (under din nuvarande graviditet) har du sökt vård för att ditt väntade barn har rört sig **mindre** eller **annorlunda**?

- ☐ Ingen gång
- ☐ 1 gång
- ☐ 2 gånger
- ☐ 3 gånger
- ☐ 4 gånger eller fler

4. Försök att beskriva **hur** ditt barn rört sig **mindre** eller **annorlunda**:

………

………

………

………

………

………

………

5. Vem **uppmanade** dig att komma för en undersökning?
Kryssa för det alternativ som var **viktigast** för att du skulle komma:

- ☐ Barnmorskan inom mödrahälsovården
- ☐ Barnmorskan på förlossningen
- ☐ Min partner
- ☐ Annan person/andra personer (vem?)……………………………………………………
- ☐ **Ingen** uppmanade mig, jag kom på **eget initiativ**

6. Varför kommer du just idag för en undersökning?

……

……

……

7. Finns det något som orsakat att du **inte kommit tidigare** för en undersökning?

……

……

……

8. Hur **länge** har du känt **minskade** eller **annorlunda** fosterrörelser?

- ☐ Kortare tid än 3 timmar
- ☐ Cirka 6 timmar
- ☐ Cirka 12 timmar
- ☐ Cirka 1 dygn
- ☐ Cirka 2 dygn
- ☐ Cirka 3 dygn
- ☐ Cirka 4 dygn till 1 vecka
- ☐ Längre tid än 1 vecka

9. Hur tycker du att rörelserna känns **nu, jämfört** med hur du upplevde rörelserna för **två veckor sedan**?

	Håller **inte alls** med	Håller med till viss del	Håller **helt** med
Jag tycker att **antalet** rörelser **minskat**			
Jag tycker att **antalet** rörelser **ökat**			
Jag tycker att **antalet** rörelser i stort sett är **samma**			
Jag tycker att **styrkan** i rörelserna **minskat**			
Jag tycker att **styrkan** i rörelserna **ökat**			
Jag tycker att **styrkan** rörelserna i stort sett är **samma**			

10. När kände du ditt barn röra sig **senast**?
(kryssa för det alternativ som bäst motsvarar det som gäller för dig)

- ☐ Cirka 1 timme sedan
- ☐ Cirka 2 timmar sedan
- ☐ Cirka 3 till 4 timmar sedan
- ☐ Cirka 5 till 6 timmar sedan
- ☐ Cirka 12 timmar sedan
- ☐ Cirka 18 timmar sedan
- ☐ Cirka 1 dygn sedan
- ☐ Cirka 2 dygn sedan
- ☐ Cirka 3 till 7 dygn sedan
- ☐ Längre än 1 vecka sedan

11. Vilka olika **typer av rörelser** har du upplevt att ditt barn gjort de **senaste två dynen**?

	Håller **inte alls** med	Håller med till viss del	Håller **helt** med
**Kraftiga rörelser:**<br>Rörelserna har varit starka och kraftfulla			
**Stretchande rörelser:**<br>Det har känts som att barnet tagit spjärn och försökte sträcka på kroppen			
**Långsamma rörelser:**<br>Rörelserna har varit långsamma			
**Sida-till-sida rörelser:**<br>Det har känts som att barnet vänt sig från sida till sida			
**Stora rörelser:**<br>Det har känts som om hela barnets kropp har rört sig			
**Lätta rörelser:**<br>Rörelserna har varit svaga			
**Ryckiga rörelser:**<br>Rörelserna har känts som ryckningar			
**Hicka:**<br>Barnet hickade			
**Mycket kraftiga snabba rörelser vid ett tillfälle:**<br>Barnet har varit extremt aktivt en kort stund för att sedan bli stilla			
Jag har **inte känt några rörelser** från mitt barn **senaste två dygnen**			
Beskriv med egna ord vad du känt **senaste två dygnen:**			

12. Har du känt **sammandragningar** den **senaste månaden**?

	**Ingen** gång	**Varje vecka**	**Varje dag**
Kraftiga sammandragningar			
Lätta sammandragningar			
Regelbundna sammandragningar			
Oregelbundna sammandragningar			

13. Hur har du uppmärksammat ditt barns rörelser **den senaste månaden**?

	**Ingen** gång	**Varje vecka**	**Varje dag**
Jag har **koncentrerat** mig på barnets rörelser en stund			
Jag har **räknat** antalet rörelser en viss tid (exempelvis 10-15 minuter)			
Jag har noterat **hur lång tid** det tog för mitt barn att röra sig tio gånger			

14. Hur **tydligt** har du känt ditt barn under **den senaste månaden**?
Kryssa i det påstående som gäller för dig.

	Håller **inte alls** med	Håller med till viss del	Håller **helt** med
Jag har känt mitt barn tydligt			
Jag har haft svårt att känna när mitt barn är vaket			
Jag har haft lätt att känna när mitt barn var vaket			
Mitt barn har rört sig svagt hela månaden			

15. Hur brukar du **vanligtvis** lägga dig när du ska sova?

- ☐ På rygg
- ☐ På höger sida
- ☐ På vänster sida

16. Hur lade du dig att sova den **senaste natten?**

- ☐ På rygg
- ☐ På höger sida
- ☐ På vänster sida

17. Vilken är din högsta utbildningsnivå?

- ☐ Grundskola
- ☐ Gymnasium eller motsvarande
- ☐ Universitet eller högskola 1-3år
- ☐ Universitet eller högskola >3år

18. I vilket land är du född?

- ☐ Sverige
- ☐ Norden (ej Sverige)
- ☐ Europa (ej Norden), land:………………………………
- ☐ Asien, land:……………………………………………………
- ☐ Afrika, land:…………………………………………………
- ☐ Sydamerika, land:…………………………………………
- ☐ Nordamerika/Canada
- ☐ Australien/Nya Zeeland

19. Ditt personnummer (år-månad-dag- och dina 4 sista siffror) …………………………
(Vi behöver dessa uppgifter för att kunna följa upp information efter att ditt barn är fött)

20. Datum när du besvarar frågeformuläret (år-månad-dag)……………………………………

21. Här kan du skriva om det är något som du **vill förmedla till den personal** som möter gravida kvinnor som känner minskade eller annorlunda fosterrörelser?

……

……

……

……

……

22. Här kan du skriva om det är något du **vill förmedla till gravida kvinnor** som känner minskade eller annorlunda fosterrörelser?

……

……

……

……

……

Tack för din medverkan!

Lägg det ifyllda formuläret i kuvertet och klistra igen. Ge kuvertet till den barnmorska eller läkare som du träffar på sjukhuset.

Kontaktuppgifter till forskargruppen finner du på det informationsbrev om studien som du fick i samband med att du bestämde dig för att delta i undersökningen.
