# Supplementary material for: Increased labor induction and women presenting with decreased or altered fetal movements - a population-based survey
Source: PLoS One. 2019 May 2;14(5):e0216216. doi: 10.1371/journal.pone.0216216 (PMC6497262; doi:10.1371/journal.pone.0216216)
Supplement: S2 File — (PDF) [file pone.0216216.s002.pdf]

Fetal movement after gestational week 28

1. Are you seeking health care because you have felt that your baby has reduced movement or a change in movement?

- ☐ Yes
- ☐ No, I am seeking health care for ……………………….

2. What is the expected date of birth for your baby? (year-month-day)?..............

3. How many times previously (during your present pregnancy) have you sought health care because of reduced movement or changes in movement?

- ☐ None
- ☐ 1 time
- ☐ 2 times
- ☐ 3 times
- ☐ 4 or more times

4. Try to describe **how** your baby has moved less or had changes in movement:

……

……

……

……

……

……

……

……

5. Who **recommended** you to come to the clinic?
Tick one of the boxes below for the alternative that best applies to you:

- ☐ The midwife at the antenatal care center
- ☐ The midwife at the labor ward
- ☐ My partner
- ☐ Another individual/other people (who?)
- ☐ No one, I have come on my own initiative

6. Why, specifically, do you come to the clinic **today**?

………

………

………

7. Are there any reasons why you did not come to the clinic earlier?

………

………

………

8. How **long** have you felt a reduction or change in fetal movements?

- ☐ Less than 3 hours
- ☐ About 6 hours
- ☐ About 12 hours
- ☐ About 1 day
- ☐ About 2 days
- ☐ About 3 days
- ☐ About 4 days to 1 week
- ☐ More than 1 week

9. How do you feel the movements **now, compared** to how you felt the movements over the **past two weeks**?

	Do **not agree** at all	Agree in part	Agree **completely**
I think that the **number** of movements has **decreased**			
I think that **the number** of movements has **increased**			
I think that **the number** of movements is about the **same**			
I think that **the strength** of the movements **has decreased**			
I think that **the strength** of the movements **has increased**			
I think that **the strength o**f the movements is about the **same**			

10. When did you last feel your baby moving?
(Tick one of the boxes below for the alternative that best applies to you)

- ☐ About 1 hour ago
- ☐ About 2 hours ago
- ☐ About 3 to 4 hours ago
- ☐ About 5 to 6 hours ago
- ☐ About 12 hours ago
- ☐ About 18 hours ago
- ☐ About 1 day ago
- ☐ About 2 days ago
- ☐ About 3 to 7 days ago
- ☐ More than 1 week ago

11. What **type of fetal movements** have you experienced over the **past two days**?

	Do **not agree** at all	Agree in part	Agree **completely**
**Powerful movements**:<br>The movements felt strong and powerful			
**Stretching movements**:<br>The movements felt as if the baby braced itself and then tried to stretch out			
**Slow movements**:<br>The movements felt determined and slow			
**Side-to-side movements**:<br>It felt as though the baby was turning from side to side			
**Big movements**:<br>The movements felt really big as if the baby's entire body was moving			
**Light movements**:<br>The movements felt weak			
**Jerky movements**:<br>The movements felt like twitching			
**Hiccups**:<br>It felt as though the baby was hiccupping			
**One episode of very strong and rapid movements**:<br>It felt as the baby was extremely active for a short time and then become motionless			
I have **not felt any movements** from my baby during **the past two days**			
Describe what you have felt during **the past two days**:			

12. Have you experienced **contractions** during **the past month?**

	Never	About **every week**	About **every day**
Powerful contractions			
Light contractions			
Regular contractions			
Irregular contractions			

13. How have you observed your baby's movements during **the past month**?

	Never	About **every week**	About **every day**
I have tried to **concentrate** on the baby's movements for a moment			
I have **counted** the number of movements during a specific time (for example 10-15 minutes)			
I have checked the length of time it takes for my baby to move ten times			

14. How have you explicitly felt your baby during **the past month**?
Tick the box for the statement that best applies to you.

	Do **not** agree at all	Agree in part	Agree **completely**
I distinctly felt my baby			
I had difficulty telling when my baby was awake			
It was easy for me to know when my baby was awake			
My baby moved weakly throughout the month			

15. Which side do you **usually** lie on in bed when you are going to go to sleep?

- ☐ On my back
- ☐ On my right side
- ☐ On my left side

16. Which side did you lie on when you were going to sleep **last night**?

- ☐ On my back
- ☐ On my right side
- ☐ On my left side

17. What is your highest level of education?:
- ☐ Primary school
- ☐ High school or equivalent
- ☐ University or college 1-3 years
- ☐ University or college > 3 years

18. In what country were you born?

- ☐ Sweden
- ☐ Scandinavia (not Sweden)
- ☐ Europe (not Scandinavia), country:……………………………………………………………………
- ☐ Asia, country:…………………………………………………………………………………………………
- ☐ Africa, country:……………………………………………………………………………………………
- ☐ South America, country:……………………………………………………………………………………
- ☐ North America/Canada
- ☐ Australia/New Zealand

19. Your personal identity number (year-month-day- and the last 4 digits)
(We require this information in order to provide a follow up after your baby is born)

………

20. The date on which you completed the questionnaire (year-month-day)……………………

21. Write freely here, your thoughts and comments, to the health care professionals working with pregnant women who feel a reduction or change in their baby's movements.

……

……

……

……

……

22. Have you any advice to give to other pregnant women who may feel a reduction or change in their baby's movements?

……

……

……

……

……

Thanks for your participation!

Please place the completed questionnaire in the provided envelope and seal it, give the envelope to the midwife or physician you meet at the hospital.

If you have any comments, questions or suggestions, you are very welcome to contact the research group. The contact information is available in the introduction letter you received.
